# Rapid and sensitive diagnoses of dry root rot pathogen of chickpea (*Rhizoctonia bataticola* (Taub.) Butler) using loop-mediated isothermal amplification assay

**DOI:** 10.1038/srep42737

**Published:** 2017-02-20

**Authors:** Raju Ghosh, Avijit Tarafdar, Mamta Sharma

**Affiliations:** 1 International Crops Research Institute for the Semi-Arid Tropics (ICRISAT), Patancheru, Hyderabad, Telangana 502324, India

## Abstract

Dry root rot (DRR) caused by the fungus *Rhizoctonia bataticola* (Taub.) Butler, is an emerging disease in chickpea. The disease is often mistaken with other root rots like *Fusarium* wilt, collar rot and black root rot in chickpea. Therefore, its timely and specific detection is important. Current detection protocols are either based on mycological methods or on protocols involving DNA amplification by polymerase chain reaction (PCR). Here we report the rapid and specific detection of *R. bataticola* using loop-mediated isothermal amplification (LAMP) assay targeting fungal specific 5.8S rDNA sequence for visual detection of *R. bataticola*. The reaction was optimized at 63 °C for 75 min using minimum 10 fg of DNA. After adding SYBR Green I in LAMP products, the amplification was found to be highly specific in all the 94 isolates of *R. bataticola* collected from diverse geographical regions as well as DRR infected plants and sick soil. No reaction was found in other pathogenic fungi infecting chickpea (*Fusarium oxysporum* f. sp. *ciceris, Rhizoctonia solani, Sclerotium rolfsii* and *Fusarium solani*) and pigeonpea (*Fusarium udum* and *Phytophthora cajani*). The standardised LAMP assay with its simplicity, rapidity and specificity is very useful for the visual detection of this emerging disease in chickpea.

Dry root rot (DRR) caused by soil borne necrotrophic fungus *Rhizoctonia bataticola* (Taub.) Butler [Synonyms: *Macrophomina phaseolina* (Tassi) Goid] is an emerging disease in chickpea (*Cicer arietinum* L.)^1^. The DRR is more dominant when the crop is exposed to moisture stress conditions^2^ and can cause 50 to 100% yield loss under favourable conditions^3^. In recent years, *Rhizoctonia bataticola* is becoming more prevalent in agricultural areas where climate change is leading to higher temperatures. It is reported that *R. bataticola* can infect more than 284 plant species including monocot and dicot plant hosts^4^. Due to availability of wide range of natural host, *R. bataticola* can easily sustain in the dry climatic area and persist in soil for prolonged period even after rotation of the crops.

In chickpea, DRR is often mistaken with *Fusarium* wilt and other root rot diseases (collar rot, black root rot etc.), as the general symptoms of these diseases are nearly similar and visually undistinguishable in field conditions^1^. In all the cases, affected plants show foliar chlorosis and ultimately cause plant collapse. Therefore, there is a real need of an advance rapid, reliable and easy detection method for diagnosis of *R. bataticola* for better management of DRR. In recent years, PCR based methods like conventional PCR and real time PCR is being employed to detect fungal species and other microorganism^5^,^6^,^7^, but it is not cost effective and need high-quality DNA due to the effects of inhibitors on PCR sensitivity^8^,^9^. Also molecular expertise is required for true diagnosis of pathogens. Now a days, Loop-mediated isothermal amplification (LAMP) has been developed as an alternative and reliable method for the detection of microbial pathogens and diagnosis of plant diseases^10^,^11^,^12^,^13^,^14^. The advantages and simplicity of LAMP assay is that the reaction could be easily judged as positive or negative by naked eye through assessing of increased turbidity or colour change^15^,^16^, and for that it does not require any expensive instruments like thermal cycler.

The LAMP is highly sensitive, less time-consuming than conventional PCR-based methods, and less prone to inhibition from DNA preparations^17^. Reliability of primer sets and DNA sequences of interest are the most important factors in development of molecular detection of targeted organisms. The internal transcribed spacer (ITS) region of nuclear rRNA genes is suitable targets for species diversity analysis within the fungal communities^18^,^19^,^20^. The characteristic of high sequence variability within the ITS region makes itself a valuable and ideal target for developing of genus and species specific PCR primers to identify an organism. Since LAMP assay has been reported to be very useful for quick detection and identification of a broad range of microorganisms, including viruses^21^, bacteria^8^, and fungi^10^,^11^, the present study was proposed to develop highly specific and very sensitive LAMP assay for the detection of *R. bataticola* from infected plants and soil.

## Materials and Methods

### Materials studied

#### Fungal strains

A total 94 isolates of *R. bataticola* representing different chickpea growing geographical region of India were taken in this study. Other major fungal strains infecting chickpea (e.g. *Fusarium oxysporum* f. sp. *ciceris, Rhizoctonia solani, Sclerotium rolfsii* and *Fusarium solani*) and other legume crop pigeonpea (*Fusarium udum* and *Phytophthora cajani*) were taken for validation of studies (Table 1).

#### Plant and soil samples

Healthy and infected DRR chickpea plant samples were collected from greenhouse and experimental fields of International Crops Research Institute for the Semi-Arid Tropics (ICRISAT), Patancheru, Telangana State, India. The soil samples taken in this study were collected from screening plot for *R. bataticola* and from rhizosphere of DRR infected chickpea plants in field (Table 1).

#### DNA extraction

Total genomic DNA (gDNA) was isolated from all the fungal isolates and DRR infected plants using PureLink Plant Total DNA Purification kit (Invitogen, USA) as per manufacturer’s protocol. About 100 mg of frozen mycelial tissue/plant tissue was grounded in liquid N_2_ and resuspended in 250 μL Resuspension Buffer (supplied in the kit). Total gDNA was eluted in 50 μL of nuclease free water and stored at −20 °C for further downstream application. The soil DNA was extracted from 100 mg of *R. bataticola* sick soil and DRR infected chickpea rhizospheric soil using SoilMaster™ DNA Extraction Kit (Epicentre, USA) according to the manufacturer’s protocol. The obtained DNA was suspended in 200 μL of elution buffer. The purified DNA was evaluated in 0.8% agarose gel as well as by UV spectrophotometry.

### Primer design

**Figure 1:**
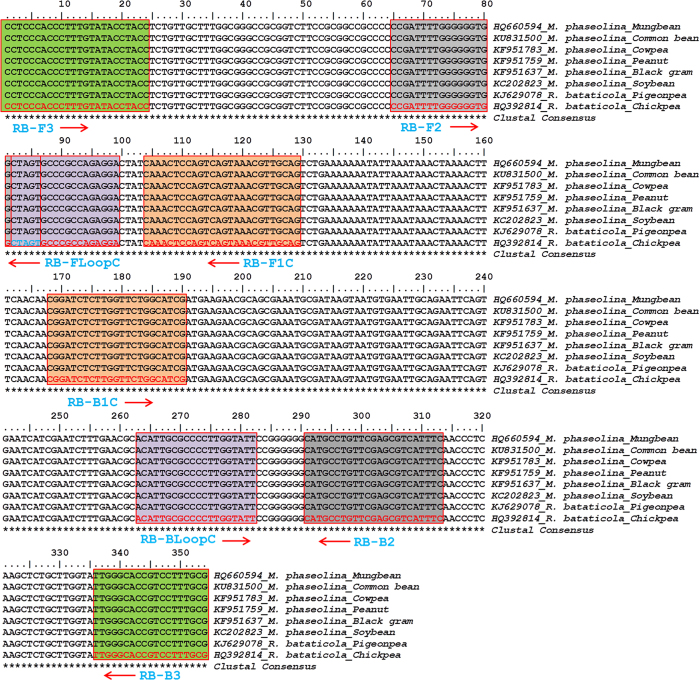
Nucleotide sequence alignment of partial ITS and 5.8S rDNA region of *Rhizoctonia bataticola* and *Macrophomina phaseolina* of different legumes. The shades sequences in different colour indicate the regions of primer development for LAMP assay. The primer sequences are specified by red colour and arrows are the direction of amplification.

As *R. bataticola* is an important plant pathogen with a broad host range and causes disease in diverse commercial crops, primers for the LAMP assay were designed from the conserved region in the partial ITS and 5.8S rRNA sequences of *R. bataticola* and *M. phaseolina* identified by multiple sequence alignment of representative isolates from different crops (Table 2 and Fig. 1). A set of six primers for LAMP assay, comprising two outer primers (RB_F3 and RB_B3), two innermost primers (RB_FIP and RB_BIP), and two loop primers (RB_LoopF and RB_LoopB) were designed manually by following the directions of Torres *et al*.^22^. The FIP was made with the complementary sequence of F1 (F1c) and F2, and the BIP made with the complementary sequence of R1 (B1c) and B2 (Table 3). The organization and position of the LAMP primers and their complementarity to target DNA used in this study are shown in Fig. 1. The designed primers were nBLAST searched on NCBI for analysing the sequence specificity and to get chance of cross reaction with other sibling species if environmental samples will assay in LAMP reaction (Table 4). To avoid the false positive reaction by cross reactivity within the primers, the secondary structures (hairpin, self-dimer and hetero-dimer) of the primers and their corresponding stability with ΔG were reviewed^23^,^24^ in OligoAnalyzer 3.1 tool (http://eu.idtdna.com/calc/analyzer) and were determined (Table 5). The universal primer pair ITS1 (5′-TCCGTAGGTGAACCTGCGG-3′) and ITS4 (5′-TCCTCCGCTTATTGATATGC-3′) and RB_F3 and RB_B3 were used for conventional PCR.

### LAMP reaction

The constituents of the LAMP assay were optimized using total gDNA extracted from *R. bataticola* fungal culture (positive control) and two negative controls (without DNA and DNA from a healthy chickpea plant). The LAMP reaction was performed in a 25 μL volume contained 2.0 μL primer mixture (20 μM each of FIP, BIP, Loop F, and Loop B primers, and 2.5 μM each of F3 and B3 primers), 1 mM dNTPs, 4 mM MgCl_2_, 2.5 μL of 10X ThermoPol Reaction Buffer (1X reaction buffer (pH 8.8) contained 20 mM Tris–HCl, 10 mM KCl, 10 mM (NH_4_)_2_SO_4_, 2 mM MgSO_4_, 0.1% Triton X-100), 8 U of *Bst* DNA polymerase (NEB, UK), 1.5 μL target DNA (about 100 ng). After completing the isothermal amplification 1 μL of SYBR Green I (Invitrogen, USA) was added for visual assay of the amplification. The reaction was carried out at 63 °C for 60 min followed by incubation at 80 °C for 10 min to inactivate the *Bst* DNA polymerase. The reactions were performed in a 0.5 mL microcentrifuge in a water bath for temperature control.

#### Optimization of LAMP reaction condition

To optimize the LAMP reaction conditions, the reaction was carried out at different temperatures, 57 °C, 60 °C, 63 °C, 66 °C and 69 °C using a gradient thermo cycler (ARKTIK Thermal Cycler, Thermo Scientific, USA). The LAMP reaction mixture was incubated at the different temperatures for different time periods, 15 min, 30 min, 45 min, 60 min, 75 min and 90 min to optimize the temperature and time/duration for the LAMP reaction. The reactions were terminated by heat inactivation at 80 °C for 10 min. The LAMP reaction was assessed visually based on colour change after adding SYBR Green I and under UV light. The LAMP amplified products were then analysed by 2% agarose gel electrophoresis.

#### Specificity of the LAMP

To determine the specificity of LAMP assay, the reaction was carried out with the extracted DNA from other legume infecting pathogenic fungi discussed previously in fungal strain section. The LAMP assay was done as described earlier at optimized temperature and time duration. The assays were visualized based on SYBR Green I colour change and then by 2% agarose gel electrophoresis. Individual fungal sample was tested by three replications and the experiment was repeated three times.

#### Sensitivity of the LAMP method

The sensitivity of the LAMP assay was determined using 10 fold serially diluted *R. bataticola* DNA ranged from 10 ng to 0.1 fg. Reaction mixture without DNA template was included as a negative control. The LAMP amplification products were analysed visually by addition of 1 μL SYBR Green I under UV light or by 2% agarose gel electrophoresis. To compare the sensitivities and specificities between LAMP assay and PCR; PCR was performed with the extracted DNA from all fungal isolates using ITS primers (Table 2). The PCR reaction was carried using the protocol detailed in Ghosh *et al*. (2015). The PCR amplified products were then analysed in 1% agarose gel stained with ethidium bromide. The experiment was repeated thrice. The detection limit in LAMP assay was defined as the last dilution with positive reaction.

#### Validation of the LAMP assay

To validate the LAMP assay, the DNA from DRR infected chickpea plants and soil samples were tested for the presence of *R. bataticola*. Based on colour change and gel electrophoresis as described above, the LAMP reaction was visualised. The experiment was carried out in triplicate and samples were considered as positive if two of the replicates showed positive response.

## Results

### Selection of LAMP primers

All *R. bataticola* isolates were identified by amplifying and sequencing of approximately 544 bp of partial ITS and 5.8S region using universal primers, ITS1 and ITS4 (Table 1). For designing of LAMP assay primers for *R. bataticola*, the conserve region of the partial ITS and 5.8S sequences was identified by multiple sequence alignment of corresponding nucleotide sequences of representative isolates in different crops (Table 2) and a set of six primers were designed (Table 3). The primers were analysed *in silico* against the nBLAST search tool using the NCBI sequence database. The BLAST results revealed that except both parts (B1c and B2) of BIP primer all primers were significantly hits the target sequences of *R. bataticola* and *M. phaseolina* with 100% identity (Table 4), although the sequence of BIP primer is 100% similar with the target sequences (Fig. 1). The hit also resulted in 100% similarity with other non-pathogenic microbes of chickpea (Table 4). During the design of LAMP primers, ΔG values were determined and the values were fixed around −9 kcal/mole or more positive than −9 kcal/mole (Table 5). For hairpins, the Tm of the hairpin was kept below of 50 °C and lower than the annealing temperature for the LAMP reaction as the strongest hairpin should be at least 50 °C (Table 5).

### Optimization of LAMP assay

**Figure 2:**
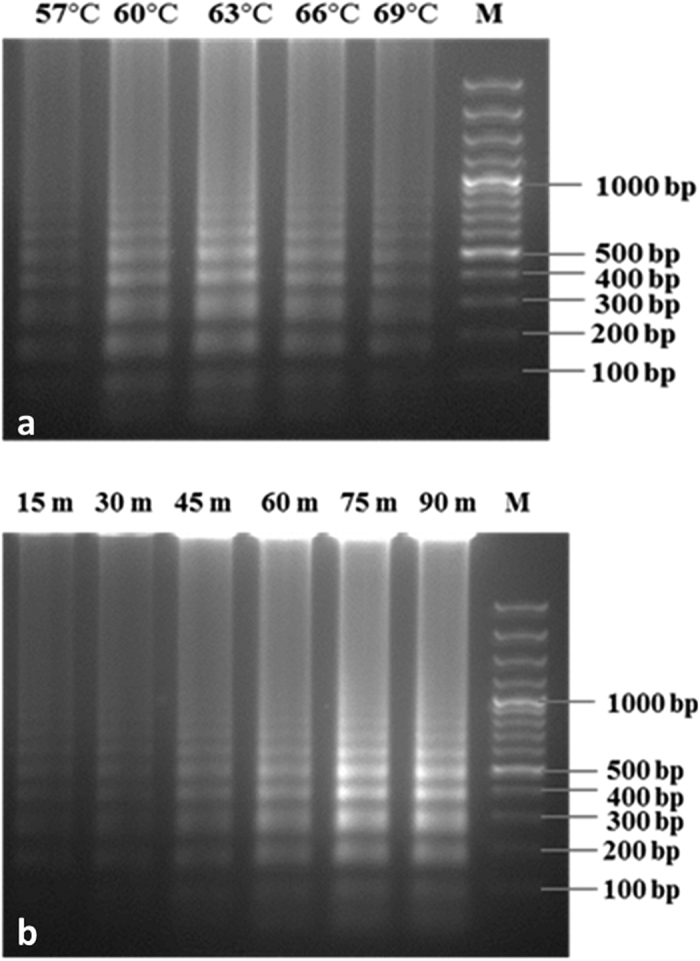
Optimization of LAMP reaction for detection of *Rhizoctonia bataticola*. (**a**) Optimization of temperature of LAMP. M GeneRuler^TM^ 100 bp Plus DNA Ladder; lanes 57 °C–69 °C indicated the reaction temperatures of LAMP. (**b**) Optimization of time duration of LAMP. M GeneRulerTM 100 bp Plus DNA Ladder; lanes 15 m–90 m indicated the reaction time duration of LAMP. All the products were detected on the basis of 2% agarose gel electrophoresis.

To determine the optimum temperature and reaction time of LAMP assay, the assay was tested within a wide range of temperatures (57 °C–69 °C) and time (15 min–90 min) using pure DNA of *R. bataticola* culture. The assay showed positive reaction at all temperatures, whether assessment was based on visual fluorescence detection or in gel electrophoresis. The characteristic ladder like bands were evident in the gel when the reaction was positive, but not if the reaction was negative. However, the high intense ladder-like band pattern was obtained in gel electrophoresis at 63 °C (Fig. 2a). Fluorescence detection result was consistent with the results from 2% agarose gel electrophoresis. For optimizing time, the LAMP reaction was conducted at 63 °C for various time duration mentioned above. The positive reaction was found in all the time duration, but strong band pattern was observed after 75 min of reaction (Fig. 2b). Therefore, 63 °C for 75 min is the optimized temperature and time for LAMP reaction for detection of DRR pathogen.

### LAMP assay specificity

**Figure 3:**
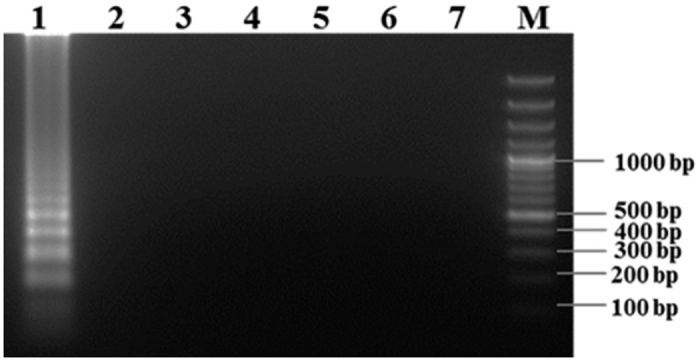
Specificity of LAMP assay for detection of *Rhizoctonia bataticola*. Approximately 10 ng of fungal genomic DNA was used in LAMP reaction. M GeneRuler^TM^ 100 bp Plus DNA Ladder; lane 1–7 DNA of *Rhizoctonia bataticola, Fusarium oxysporum* f. sp. *ciceris, Rhizoctonia solani, Sclerotium rolfsii, Fusarium solani, Fusarium udum* and *Phytophthora cajani*, respectively.

LAMP specificity was examined using DNA templates extracted from total 94 *R. bataticola* isolates collected from diverse geographical region in India. Six pathogenic wilt and root rot pathogens viz., *Fusarium oxysporum* f. sp. *ciceris, Rhizoctonia solani, Sclerotium rolfsii, Fusarium solani, Fusarium udum* and *Phytophthora cajani* infecting chickpea and pigeonpea were also taken for testing specificity (Table 1). DNA template of *R. bataticola* isolates gave positive reaction, whereas no amplification was observed for the other fungal species after incubation at 63 °C for 75 min. The LAMP reaction was assessed using 2% agarose (Fig. 3) and SYBR Green I visualisation. The result indicated that the LAMP assay developed in this study is highly specific to *R. bataticola*. Furthermore, to confirm the specificity of the LAMP primers, a PCR was carried out using primer pair, RB_F3 and RB_B3. All the *R. bataticola* isolates were amplified with a unique DNA fragment of 354 bp. However, these primers did not produce any band when DNA templates from other pathogenic fungi were used. However, primer pair ITS1 and ITS4 gave amplification of expected size of DNA fragment (Table 1).

### Sensitivity of LAMP assay

**Figure 4:**
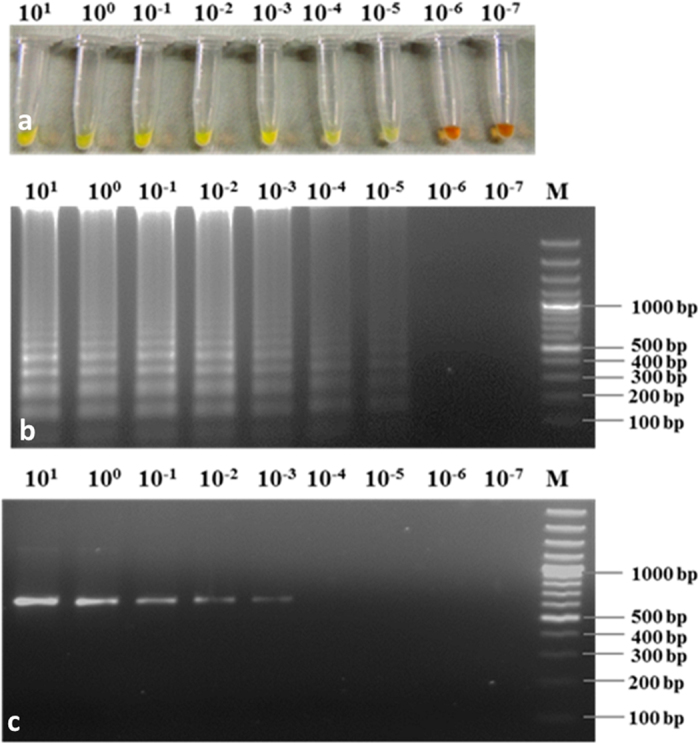
Sensitivity of LAMP assay vs. conventional PCR for detection of *Rhizoctonia bataticola* using similar concentration of DNA template. (**a**) Visual assessment of LAMP assay using SYBR Green I. (**b**) LAMP assay on the basis of 2% agarose gel electrophoresis. (**c**) Result of conventional PCR using ITS1 and ITS4 primers. M GeneRuler^TM^ 100 bp Plus DNA Ladder; lane 10^1^–10^−7^ indicated the DNA concentration in LAMP reaction starting from 10 ng (10^1^ ng) to subsequent 10 fold diluted DNA up to 0.1 fg (10^−7^ ng), respectively.

To determine the detection limit, the sensitivity of the LAMP reaction was assessed using 10-fold serially diluted *R. bataticola* DNA template. The amplicons were detected in both, by visual assessment using SYBR Green I (Fig. 4a) and 2% agarose gel electrophoresis (Fig. 4b). No positive signal was produced when less than 10 fg DNA of *R. bataticola* was used in LAMP reaction, indicating the potential detection limit of *R. bataticola* up to 10 fg of DNA. It was noticed that the amplified DNA fragments were slightly faint in 10 fg than those produced by a less diluted DNA (>10 fg). On the other hand, when same amount of DNA was used in conventional PCR, no such amplification was obtained after 1.0 pg of dilution (Fig. 4c). Results of visual detection correlated with agarose gel electrophoresis.

### Evaluation of the LAMP assays with plant and soil sample

**Figure 5:**
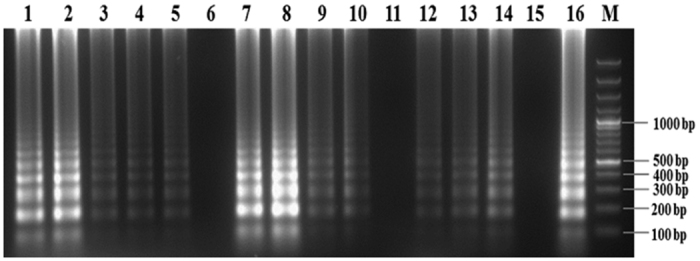
Evaluation of the LAMP assay for detection of *Rhizoctonia bataticola* in optimized conditions (63 °C for 75 min) using DNA template from infected chickpea plants and rhizospheric soil. M GeneRuler^TM^ 100 bp Plus DNA Ladder; lane 1–5 Dry root rot infected chickpea plants sampled from different fields; 6 Healthy chickpea plant from field; 7–10*R. bataticola* inoculated chickpea plants sampled from green house; 11 healthy chickpea plant from greenhouse; 12 Rhizospheric black soil from field; 13 Rhizospheric red soil from field; 14 Sick soil from greenhouse; 15 Negative control without any DNA template; 16 Positive control with genomic DNA of *R. bataticola*.

To validate the applicability of the LAMP assay at field level, the developed LAMP assay was evaluated with *R. bataticola* infested chickpea plants and soil samples as well. The plants showing typical symptom of DRR were collected from different experimental fields of ICRISAT. The LAMP assay was carried out at optimised condition to detect the presence of *R. bataticola* in tested samples. The positive LAMP reaction was found when DNA templates from the *R. bataticola* infected chickpea plants were assessed, and the products turned green in colour with SYBR Green I. Moreover, none of the DNA template from healthy plants gave positive signals, and remained orange in colour. Furthermore, the LAMP assay conducted with the DNA from rhizospheric soil of DRR infected chickpea plants as well as *R. bataticola* inoculated sick soil was also found to be positive (Fig. 5). The LAMP result was consistent when the assays were repeated. Results were consistent with PCR method.

## Discussion

In this report, we have demonstrated and optimized the LAMP for the detection of *R. bataticola*, the fungus that cause DRR disease in chickpea. LAMP assay reported here is rapid, highly sensitive, less time-consuming than conventional PCR-based DNA amplification method. It has been applied for the detection of a wide range of microorganisms including viral^13^,^25^, bacterial^26^,^27^, phytoplasma^28^, mycoplasma^29^, fungal^10^,^11^,^12^ and parasitic agents^30^.

In our study, we used SYBR Green I in LAMP assay as florescent dye which is non-mutagenic and eco-friendly in nature, as the replacement of other potential human mutagen florescent dyes like ethidium bromide. Subsequently, uses of *Bst* DNA polymerase in LAMP reaction, it permits to strand displacement DNA synthesis and the reactions can be performed under isothermal conditions using a simple incubator, such as a water bath or heating block. With taking these advantages, LAMP detection technique can be employed in diagnosis of disease in field level even also in remote area where the laboratories are not well equipped.

We validated our developed LAMP assay using the primers generated from partial ITS and 5.8S rDNA gene for quick detection of *R. bataticola* from varied samples viz. fungal culture, diseased plants and soil samples. The highly sensitive and variable ITS region is idle target rather than other single-copy genes in genomic DNA to sufficient discriminate of some closely related fungal species^31^,^32^ because of its presence in 100 or more copies in the fungal genome and has competence to give amplification from a very small number of micro-organisms^33^. Then again, to avoid secondary structures in primers with abundant G-C bonds e.g. hairpins, self-dimer and heterodimer, the ΔG of the primers were kept more positive than −9 kcal/mole or very less negative than the same value^23^,^24^ as those can give false positive reaction in assay. When the LAMP primers were analysed *in silico* for its specificity, it showed significant hit with our target organisms *R. bataticola* and *M. phaseolina* in nBLAST. For confirming the specificity of our developed LAMP primers, we assayed those primers with DNA isolated from 94 *R. bataticola* isolates along with six other plant pathogenic fungi. During this LAMP assay, except all *R. bataticola* DNA, no colour change was obtained with DNA of other six pathogenic fungus. Thus, this result indicated that the designed primers and LAMP assay were highly specific for *R. bataticola*, as it correctly distinguished between *R. bataticola* and the other pathogens. The reaction mixture without DNA also showed no change in colour during LAMP assay. Previously, the primers from rDNA-ITS gene have been used to successfully detect *Pythium aphanidermatum* in infected tomatoes^34^ and *Phytophthora capsici* in infected peppers, tomatoes, and other agronomic and ornamental crops of the Solanaceae and Cucurbitaceae families^35^.

The detection limit in our study was found to be 10 fg DNA of *R. bataticola*, below this level no colour change was noticed. This detection limit was lower than previously reported LAMP methods used to detect other fungal pathogens e.g. *Sclerotinia sclerotiorum*^12^, *Phytophthora sojae*^36^, *P. ramorum* and *P. kernoviae*^37^, indicating greater sensitivity. Comparison of LAMP assay with the conventional PCR showed the LAMP assay using SYBR Green I dye significantly improved the detection efficiency of *R. bataticola*. This result of LAMP assay was significant and concordant with the reports published previously for the detection of some plant pathogens^12^,^36^,^37^.

Positive reaction in LAMP assay with the DNA isolated from *R. bataticola* infected chickpea plants sampled from field, further validated our results. DNA from healthy plants gave no reaction. It has previously been used in detection/screening of plant pathogens like *Phytophthora capsici*^35^, *P. ramorum*^38^, *Pythium aphanidermatum*^34^, *Meloidogyne enterolobii*^39^ from infected plants in field and soil samples. In this study, we have designed LAMP primers using sequence of *R. bataticola/M. phaseolina* isolates infecting chickpea as well as other crops from worldwide and *in silico* analyses of ITS sequences showed low genetic diversity within the Indian and global isolates, hence the utility of our developed LAMP assay will be equally useful for detection of *R. bataticola* isolates in any other crops globally. In future, LAMP diagnostic kit will be very useful for monitoring the disease complex in the fields, further helpful in developing the timely management strategies.

## Additional Information

**How to cite this article**: Ghosh, R. *et al*. Rapid and sensitive diagnoses of dry root rot pathogen of chickpea (*Rhizoctonia bataticola* (Taub.) Butler) using loop-mediated isothermal amplification assay. *Sci. Rep.* **7**, 42737; doi: 10.1038/srep42737 (2017).

**Publisher's note:** Springer Nature remains neutral with regard to jurisdictional claims in published maps and institutional affiliations.

## Figures and Tables

**Table 1:** Details of thee fungal isolates, plant and soil samples used in the LAMP detection assay.

Samples	Collection site	State	LAMP detection		Conventional PCR	
			Florescence	Agarose gel	RB_F3/RB_B3	ITS1/ITS4
*R. bataticola* isolates (chickpea)						
RB1	Kanpur	Uttar Pradesh	+	+	+	+
RB2	Coimbatore	Tamil Nadu	+	+	+	+
RB3	ICRISAT, BIL 01	Andhra Pradesh	+	+	+	+
RB4	ICRISAT, BIL 01	Andhra Pradesh	+	+	+	+
RB5	ICRISAT, BP 4	Andhra Pradesh	+	+	+	+
RB6	ICRISAT, BP 10	Andhra Pradesh	+	+	+	+
RB7	ICRISAT, BIL 01	Andhra Pradesh	+	+	+	+
RB8	ICRISAT, BUS 03	Andhra Pradesh	+	+	+	+
RB9	ICRISAT, BR 05	Andhra Pradesh	+	+	+	+
RB10	ICRISAT, BIL 02	Andhra Pradesh	+	+	+	+
RB11	ICRISAT, BIL 02	Andhra Pradesh	+	+	+	+
RB12	Pati	Andhra Pradesh	+	+	+	+
RB13	ICRISAT, BP 02	Andhra Pradesh	+	+	+	+
RB14	Jodhpur	Madhya Pradesh	+	+	+	+
RB15	Jabalpur	Madhya Pradesh	+	+	+	+
RB16	Delhi	Delhi	+	+	+	+
RB17	Damoh 1	Madhya Pradesh	+	+	+	+
RB18	Damoh 2	Madhya Pradesh	+	+	+	+
RB19	Damoh 3	Madhya Pradesh	+	+	+	+
RB20	Damoh 4	Madhya Pradesh	+	+	+	+
RB21	Damoh 5	Madhya Pradesh	+	+	+	+
RB22	Damoh 6	Madhya Pradesh	+	+	+	+
RB23	Damoh 7	Madhya Pradesh	+	+	+	+
RB24	ICRISAT, BUS 04	Andhra Pradesh	+	+	+	+
RB25	ICRISAT, BUS 07	Andhra Pradesh	+	+	+	+
RB26	ICRISAT, BIL 02	Andhra Pradesh	+	+	+	+
RB27	ICRISAT, BUS 07	Andhra Pradesh	+	+	+	+
RB28	ICRISAT, BR 04H	Andhra Pradesh	+	+	+	+
RB29	ICRISAT, BUS 03	Andhra Pradesh	+	+	+	+
RB30	ICRISAT, BIL 02D	Andhra Pradesh	+	+	+	+
RB31	ICRISAT, BUS 03	Andhra Pradesh	+	+	+	+
RB32	ICRISAT, BIL 05	Andhra Pradesh	+	+	+	+
RB33	ICRISAT, BP 04	Andhra Pradesh	+	+	+	+
RB34	ICRISAT, BR 05D	Andhra Pradesh	+	+	+	+
RB35	ICRISAT, BUS 03	Andhra Pradesh	+	+	+	+
RB36	ICRISAT, BR 04F	Andhra Pradesh	+	+	+	+
RB37	ICRISAT, BP 04	Andhra Pradesh	+	+	+	+
RB38	ICRISAT, BR 04F	Andhra Pradesh	+	+	+	+
RB39	ICRISAT, BM 14	Andhra Pradesh	+	+	+	+
RB40	ICRISAT, BR 04	Andhra Pradesh	+	+	+	+
RB41	ICRISAT, BP 04	Andhra Pradesh	+	+	+	+
RB42	ICRISAT, BR 04F	Andhra Pradesh	+	+	+	+
RB43	ICRISAT, BW 01A	Andhra Pradesh	+	+	+	+
RB44	ICRISAT, BIL 01	Andhra Pradesh	+	+	+	+
RB45	ICRISAT, BW 03	Andhra Pradesh	+	+	+	+
RB46	ICRISAT, BW 04	Andhra Pradesh	+	+	+	+
RB47	ICRISAT, BR 04I	Andhra Pradesh	+	+	+	+
RB48	ICRISAT, BR 04H	Andhra Pradesh	+	+	+	+
RB49	Jabalpur 1	Madhya Pradesh	+	+	+	+
RB50	Jabalpur 5	Madhya Pradesh	+	+	+	+
RB51	Jabalpur 6	Madhya Pradesh	+	+	+	+
RB52	Jabalpur 8	Madhya Pradesh	+	+	+	+
RB53	Brampuri, Damoh 8	Madhya Pradesh	+	+	+	+
RB54	Brampuri, Damoh 9	Madhya Pradesh	+	+	+	+
RB55	Katni	Madhya Pradesh	+	+	+	+
RB56	JNKVV, Jabalpur	Madhya Pradesh	+	+	+	+
RB57	Rewa	Madhya Pradesh	+	+	+	+
RB58	Bachara. Satna	Madhya Pradesh	+	+	+	+
RB59	IIPR, Kanpur	Uttar Pradesh	+	+	+	+
RB60	ICRISAT, BIL 02	Andhra Pradesh	+	+	+	+
RB61	Dhaulakuan	Himachal Pradesh	+	+	+	+
RB62	Ramnagar, Pantnagar	Uttarakhand	+	+	+	+
RB63	BAU, Ranchi	Jharkhand	+	+	+	+
RB64	ICRISAT, BP 02C	Andhra Pradesh	+	+	+	+
RB65	ICRISAT, BP 15	Andhra Pradesh	+	+	+	+
RB66	ICRISAT, BP 03B	Andhra Pradesh	+	+	+	+
RB67	ICRISAT, BP 03C	Andhra Pradesh	+	+	+	+
RB68	ICRISAT, BP 08A	Andhra Pradesh	+	+	+	+
RB69	ICRISAT, BP 08B	Andhra Pradesh	+	+	+	+
RB70	ICRISAT, BR 05D	Andhra Pradesh	+	+	+	+
RB71	ICRISAT, BR 05B	Andhra Pradesh	+	+	+	+
RB72	ICRISAT, BL 04A	Andhra Pradesh	+	+	+	+
RB73	ICRISAT, BL0 4	Andhra Pradesh	+	+	+	+
RB74	ICRISAT, BM 13	Andhra Pradesh	+	+	+	+
RB75	ICRISAT, BM 13	Andhra Pradesh	+	+	+	+
RB76	ICRISAT, BM 08C	Andhra Pradesh	+	+	+	+
RB77	ICRISAT, BW 02A	Andhra Pradesh	+	+	+	+
RB78	ICRISAT, BW 02B	Andhra Pradesh	+	+	+	+
RB79	ICRISAT, BW 02C	Andhra Pradesh	+	+	+	+
RB80	ICRISAT, BW 04A	Andhra Pradesh	+	+	+	+
RB81	ICRISAT, BW 05A	Andhra Pradesh	+	+	+	+
RB82	ICRISAT, BW 05B	Andhra Pradesh	+	+	+	+
RB83	ICRISAT, BW 08	Andhra Pradesh	+	+	+	+
RB84	ICRISAT, BIL 01	Andhra Pradesh	+	+	+	+
RB85	ICRISAT, BIL 01	Andhra Pradesh	+	+	+	+
RB86	ICRISAT, BIL 01	Andhra Pradesh	+	+	+	+
RB87	ICRISAT, BIL 03C	Andhra Pradesh	+	+	+	+
RB88	ICRISAT, BIL 03	Andhra Pradesh	+	+	+	+
RB89	ICRISAT, BIL 04	Andhra Pradesh	+	+	+	+
RB90	ICRISAT, BIL 05B	Andhra Pradesh	+	+	+	+
RB91	ICRISAT, BIL 05C	Andhra Pradesh	+	+	+	+
RB92	ICRISAT, BIL 05C	Andhra Pradesh	+	+	+	+
RB93	ICRISAT, JM 08B	Andhra Pradesh	+	+	+	+
RB94	ICRISAT, BR 05C	Andhra Pradesh	+	+	+	+
DRR infected chickpea plants (field)						
Sample 1	ICRISAT, BIL 02	Andhra Pradesh	+	+	+	+
Sample 2	ICRISAT, BW 08	Andhra Pradesh	+	+	+	+
Sample 3	ICRISAT, BM 13	Andhra Pradesh	+	+	+	+
Sample 4	ICRISAT, BP 15	Andhra Pradesh	+	+	+	+
Sample 5	ICRISAT, BIL 01	Andhra Pradesh	+	+	+	+
Healthy	ICRISAT, BIL 02	Andhra Pradesh	−	−	−	−
DRR infected chickpea plants (greenhouse)						
Sample 1	Experimental sample	ICRISAT	+	+	+	+
Sample 2	Experimental sample	ICRISAT	+	+	+	+
Sample 3	Experimental sample	ICRISAT	+	+	+	+
Sample 4	Experimental sample	ICRISAT	+	+	+	+
Healthy	Experimental sample	ICRISAT	−	−	−	−
Sick soil (DRR)						
Rhizospheric black soil	ICRISAT, BP 04	Andhra Pradesh	+	+	+	MB^a^
Rhizospheric red soil	ICRISAT, RL17	Andhra Pradesh	+	+	+	MB
Sick soil (non-rhizospheric)	Experimental sick plot	ICRISAT	+	+	+	+
Other fungal pathogens						
*F. oxysporum* f. sp. *ciceris* (chickpea)	*In vitro* culture	ICRISAT	−	−	−	+
*R. solani* (chickpea)	*In vitro* culture	ICRISAT	−	−	−	+
*S. rolfsii* (chickpea)	*In vitro* culture	ICRISAT	−	−	−	+
*F. solani* (chickpea)	*In vitro* culture	ICRISAT	−	−	−	+
*F. udum* (pigeonpea)	*In vitro* culture	ICRISAT	−	−	−	+
*P. cajani* (pigeonpea)	*In vitro* culture	ICRISAT	−	−	−	+

^a^Multiple bands.

**Table 2:** Nucleotide sequence analysis of representative *Rhizoctonia bataticola*/*Macrophomina phaseolina* isolates infecting economically important plants and their disease list.

Pathogen	Host		Disease	Acc. No.	Country source	Sequence alignment analysis for Acc. No. HQ392814 (QC/ID in per cent)*	
	Common name	Scientific name				Complete sequence	Sequence fragment taken for primer design
*Rhizoctonia bataticola*	Chickpea	*Cicer arientinum*	Dry root rot	HQ392814	India	100/99	100/100
	Pigeonpea	*Cajanus cajan*	Dry root rot	KJ629078	India	95/99	100/100
	Linseed	*Linum usitatissimum*	Wilt	KM247370	India	95/98	100/99
	Spider lily	*Crinum asiaticum*	Leaf disease	KX447538	Malaysia	99/98	100/100
*Macrophomina phaseolina*	Impatiens	*Impatiens sp.*	Root disease complex	KU726237	USA	98/99	100/100
	Sorghum	*Sorghum bicolor*	Charcoal rot	KU856652	Denmark	98/99	100/100
	Olive	*Olea europea*	Die-back	KU863545	Tunisia	98/99	100/100
	Sunflower	*Helianthus annuus*	Charcoal rot	KT862032	Mongolia	95/99	100/100
	Potato	*Solanum tuberosum*	Charcoal rot/tuber blemishes	KU721993	South Africa	85/100	100/100
	Common bean	*Phaseolus vulgaris*	Charcoal rot/leaf blight	KU831500	Tunisia	94/99	100/100
	Indian jasmine	*Jasminum multiflorum*	Root rot	KT768135	India	98/99	100/100
	Cowpea	*Vigna unguiculata*	Charcoal rot	KF951783	Senegal	92/99	100/100
	Peanut	*Arachis hypogae*	Dry root rot/charcoal rot	KF951759	Senegal	96/99	100/100
	Lady’s fingers	*Abelmoschus esculentas*	Dry root and foot rot	KF951754	Senegal	97/99	100/100
	Roselle	*Hibiscus sabdarifa*	Charcoal rot	KF951701	Louga, Senegal		
	97/99	100/100					
	Pearl millet	*Pennisetum glaucum*	Dry root rot	KF951691	Niger	92/99	100/100
	Black gram	*Phaseolus mungo*	Charcoal rot	KF951637	Denmark	93/99	100/100
	Chrysanthemum	*Chrysanthemum sp.*	Charcoal rot	KF951633	USA	92/99	100/100
	Sugarcane	*Saccharum officinarum*	Charcoal rot	KF951631	India	97/99	100/100
	Pigeonpea	*Cajanus indicus*	Dry root rot	KF951628	Sri Lanka	93/99	100/100
	Maize	*Zea mays*	Charcoal rot	KF951627	Palestine	92/99	100/100
	Sesamum	*Sesamum indicum*	Charcoal rot	KF951624	Uganda	93/99	100/100
	Derris legume	*Derris elliptica*	Charcoal rot	KF951623	Malaysia	93/99	100/100
	Black cottonwood	*Populus trichocarpa*	Charcoal rot	KF428466	USA	97/99	100/100
	Cumin	*Cuminum cyminum*	Charcoal rot	KF453968	Turkey	95/99	100/100
	Strawberry	*Fragaria sp.*	Crown rot	KC822431	China	98/99	100/100
	Pectilis orchid	*Pectilis susannae*	Charcoal rot	KC920477	India	78/100	100/100
	Soybean	*Glycin max*	Charcoal rot	KC202823	Iran	93/99	100/100
	Sweet potato	*Ipomoea batatas*	Charcoal rot	JX945170	USA	98/99	100/100
	Turmeric	*Carcuma longa*	Charcoal rot	JX535007	India	96/99	100/100
	Mungbean	*Vigna radiata*	Root rot and leaf blight	HQ660594	China	98/99	100/100
	Golden samphire	*Inula crithmoides*	Root rot	HQ649832	Spain	93/99	100/100

^*^Values of QC (quarry coverage) and identity (ID) scores of all sequence segments retrieve from NCBI nucleotide sequence database that matched the query sequence (HQ392814).

**Table 3:** Information of the primers used for the LAMP reaction.

Primers name	Sequences (5′-3′)	Type	Primer position (nt)	Length (bp)	GC (%)	Tm
RB_F3	CCTCCCACCCTTTGTATACCTACC	Forward outer	1–24	24	54.2	58.4
RB_B3	CGCAAAGGACGGTGCCCAA	Backward outer	336–354	19	63.2	61.4
RB_FIP (F1c+F2)	CTGCAACGTTTACTGACTGGAGTTTG-CCGATTTTGGGGGGTGGCTAGT	Forward inner	(104–129) + (65–86)	26 + 22	46.2–59.1	58.2–61.7
RB_BIP (B1c + B2)	CGGATCTCTTGGTTCTGGCATCG-GAAATGACGCTCGAACAGGCATG	Backward inner	(168–190) + (291–313)	23 + 23	54.2–52.2	60.9–58.8
RB_LoopF	TCCTCTGGCGGGCACTAG	Forward loop forming	82–99	18	66.7	59.4
RB_LoopB	ACATTGCGCCCCTTGGGATT	Backward loop forming	263–282	20	55	60.2

**Table 4:** *In silico* specificity and cross reactivity analysis of LAMP primers through NCBI database and details of 100 hits generated in nBLAST.

List of primers	*R. bataticola*/*M. phaseolina*		Other microbes		
	Identity (%)	Number of hits	Identity (%)	Number of hits	Name of the dominant microbes hit in primer blast (numbers in the parentheses denote the number of hits)
RB_F3	100	96	100	4	Uncultured *Helotiales* sp. (4)
RB_B3	100	40	100	60	*Botryosphaeria dothidea* (51), Uncultured fungal strains (7), *Fusicoccum fabicercianum* (2)
RB_F1c	100	100	—	—	—
RB_F2	100	100	—	—	—
RB_B1c	—	?*	100	100	Uncultured fungal strains (22), *Coniella* spp. (14), Uncultured *Helotiales* sp. (7), *Fusarium* spp. (6), *Oidiodendron* sp. (5), *Xylariaceae* sp. (5), *Botryosphaeria dothidea* (4), *Nemania* sp. (4) and remaining hits are of different microbes with single entry
RB_B2	—	?*	100	100	*Coniella* sp. (60), Uncultured fungal strains (9), *Oidiodendron* sp. (9), *Fusarium* spp. (6), *Xylariaceae* sp. (5), *Microsporum gypseum* (4), *Nemania* sp. (4) and remaining hits are of different microbes with single entry
RB_Loop_F	100	89	100	11	*Botryosphaeria mamane* (11)
RB_Loop_B	100	3	100	6	*Dothideomycetes* sp. (1), *Hormonema* sp. (1), *Saccharomyces cerevisiae* (1), *Ampelomyces quisqualis* (1), Uncultured *Heyderia* (1), Uncultured *Helotiales* (1)
	—	—	85–95	91	Uncultured fungal strains (36), *Lasiodiplodia theobromae*, (10), Russula sp. (4), *Mesorhizobium* (3), Uncultured *Helotiales* sp (2) and remaining hits are of different microbes with single entry

^*^Not hit in nBLAST analysis, but sequences are 100% similar with *R. bataticola*/*M. phaseolina* (Table 2 and Fig. 1).

**Table 5:** *In silico* hairpin, self-dimer or hetero-dimer analysis of primers used in LAMP assay.

List of primers	ΔG for hetero-dimer					ΔG for self-dimer	Hairpin	
	RB_B3	RB_FIP	RB_BIP	RB_LoopF	RB_LoopB		ΔG	Tm
RB_F3	−10.51	−12.51	−5.02	−6.14	−9.67	−6.08	0.23	22.3
RB_B3	—	−10.04	−8.16	−12.57	−10.65	−5.09	−0.59	31.6
RB_FIP	—	—	−9.73	−8.16	−9.21	−10.18	−1.75	43.0
RB_BIP	—	—	—	−6.75	−6.75	−6.76	−1.97	36.5
RB_Loop_F	—	—	—	—	−9.82	−4.16	0.3	17.1
RB_Loop_B	—	—	—	—	—	−9.89	−1.69	50.5
